# TiO_2_, SiO_2_ and ZrO_2_ Nanoparticles Synergistically Provoke Cellular Oxidative Damage in Freshwater Microalgae

**DOI:** 10.3390/nano8020095

**Published:** 2018-02-08

**Authors:** Yinghan Liu, Se Wang, Zhuang Wang, Nan Ye, Hao Fang, Degao Wang

**Affiliations:** 1 Collaborative Innovation Center of Atmospheric Environment and Equipment Technology, School of Environmental Science and Engineering, Nanjing University of Information Science and Technology, Nanjing 210044, China; yinghan_liu@163.com (Y.L.); yenan_nuist@163.com (N.Y.); fh@nuist.edu.cn (H.F.); 2 School of Environmental Science and Technology, Dalian Maritime University, Dalian 116023, China; degaowang@163.com

**Keywords:** metal-oxide nanoparticles, mixture toxicity, freshwater algae, cellular response, oxidative damage

## Abstract

Metal-based nanoparticles (NPs) are the most widely used engineered nanomaterials. The individual toxicities of metal-based NPs have been plentifully studied. However, the mixture toxicity of multiple NP systems (*n* ≥ 3) remains much less understood. Herein, the toxicity of titanium dioxide (TiO_2_) nanoparticles (NPs), silicon dioxide (SiO_2_) NPs and zirconium dioxide (ZrO_2_) NPs to unicellular freshwater algae *Scenedesmus obliquus* was investigated individually and in binary and ternary combination. Results show that the ternary combination systems of TiO_2_, SiO_2_ and ZrO_2_ NPs at a mixture concentration of 1 mg/L significantly enhanced mitochondrial membrane potential and intracellular reactive oxygen species level in the algae. Moreover, the ternary NP systems remarkably increased the activity of the antioxidant defense enzymes superoxide dismutase and catalase, together with an increase in lipid peroxidation products and small molecule metabolites. Furthermore, the observation of superficial structures of *S. obliquus* revealed obvious oxidative damage induced by the ternary mixtures. Taken together, the ternary NP systems exerted more severe oxidative stress in the algae than the individual and the binary NP systems. Thus, our findings highlight the importance of the assessment of the synergistic toxicity of multi-nanomaterial systems.

## 1. Introduction 

Engineered nanomaterials are emerging with potential extensive application owing to their unprecedented physicochemical properties [1,2]. Specially, metal-oxide nanoparticles (NPs) have been permeating in diverse fields including medicines [3], fuel [4], clothing [5] and cosmetics [6]. In the meantime, NPs may enter the environment via many routes [7], accompanied by the diversity of nanotechnology applications. Once released into the environment, NPs can undergo transformations [8] and pose toxicity to organisms [9,10] in different environmental compartments. To date, the fate and effect of NPs in the aquatic environment have become a hot research topic in eco-toxicological communication in particular.

In natural environment, organisms are exposed to a mixture comprising of two or more contaminants, instead of individual ones [11]. Recently, there are some pioneering studies that highlighted combined toxicity of binary mixtures of CeO_2_ and TiO_2_ NPs [12], CuO and ZnO NPs [13], TiO_2_ and ZnO NPs [14], SiO_2_ and TiO_2_ NPs [15]. Most of the studies stated that toxicity of joint action of binary NP mixtures to organisms is antagonistic. For example, Yu et al. [12] found that the binary toxicity (antagonistic toxicity) of CeO_2_ and TiO_2_ NPs to *Nitrosomonas europaea* was generally lower than that induced by the single NPs. Hua et al. [16] also stated that TiO_2_ NPs reduced the effects of ZnO NPs on zebrafish embryos. However, some studies addressed that binary NP mixtures have a synergistic effect on organisms. For instance, Yu et al. [12] showed that the mixture of CeO_2_ and ZnO NPs exerted higher cytotoxicity (synergistic cytotoxicity) to *N. europaea* than that from single NPs. Tsugita et al. [15] also concluded that SiO_2_ and TiO_2_ NPs synergistically induced macrophage inflammatory responses and subsequent lung inflammation. Taken together, the combined toxicity of binary NP mixtures is related with organisms and mixture systems. To the best of our knowledge, studies on the toxicity of multiple NPs such as ternary NP mixtures to organisms are scarce.

Algae, as a key primary producer, play an important role in maintaining ecological balance [17]. Because of their small size, fast breeding and toxicant sensitivity, algae could be a model organism to assess potential toxicity of NPs in the aquatic system [18,19,20,21]. It was the purpose of the present study to investigate the toxicity of TiO_2_, SiO_2_ and ZrO_2_ NPs from single to ternary NP systems to *Scenedesmus obliquus* at the cellular level. For this purpose, two main objectives were (1) to determine the physic-chemical properties of the single, binary and ternary NPs in a model freshwater; (2) to investigate the effects of single, binary and ternary NPs on the algal photosynthesis, membrane potential and permeability, reactive oxygen species (ROS) generation, as well as anti-oxidative enzyme and non-enzyme systems.

## 2. Methods

### 2.1. Test Material and Test Medium

TiO_2_ NPs with a primary size of 21 ± 5 nm (advertised specific surface area 50 ± 10 m^2^/g; purity > 99.5%), SiO_2_ NPs with a primary size of 7–14 nm (advertised specific surface area >200 m^2^/g; purity > 99.8%) and ZrO_2_ NPs with a primary size of 5–25 nm (advertised specific surface area 130 ± 20 m^2^/g; purity > 97.2%) were purchased from PlasmaChem GmbH (Berlin, Germany). The NP stock suspensions were freshly prepared in ultra-high pure water after 30 min sonication in a water bath sonicator and then stored at 4 °C until use. Algae culture medium was prepared as diluted water at pH 7.8 ± 0.2 according to OECD guideline [22].

### 2.2. Physicochemical Analysis

The NPs and the particles in the algae medium were characterized by using a super-resolution scanning electron microscope (SEM, MERLIN Compact, ZEISS, Oberkochen, Germany) and a transmission electron microscope (TEM, JOEL 2100f, JOEL Ltd., Tokyo, Japan), respectively. Zeta potential (ZP) and hydrodynamic diameters (*D*_H_) of the NP suspensions at 1 mg/L were analyzed at 0 and 96 h after incubation in the algae medium using a ZetaSizer instrument (Nano ZS90, Malvern Instruments Ltd., Worcestershire, UK). The ZP and *D*_H_ measurement were performed in three independent experiments (each time contained three parallel) and the data presented are the mean of the runs.

### 2.3. Algal Growth Assays

The unicellular freshwater algae *S. obliquus* was obtained from the Chinese Academy of Sciences Institute of Hydrobiology (Wuhan, China). Exponentially growing algae cells (with a final density of 3 × 10^5^ cells/mL) were added to control (aimed at exploring the association among test materials) and treated experiments. Internal control experiments were required in order to eliminate the absorbance effects of materials. All flasks containing various NPs were incubated in an artificial growth chamber consistently at a temperature of 24 ± 1 °C for 96 h with a photoperiod of 12-h light (3000–4000 lx) and 12-h dark. The algae were exposed to single, binary, ternary mixtures of TiO_2_, SiO_2_ and ZrO_2_ NPs. The following concentrations of nanoparticles were mainly selected: 1 mg/L and 1 μg/L of NPs alone, 1 mg/L and 1 μg/L of single NPs in binary combination and 1 mg/L and 1 μg/L of single NPs in ternary combination. 1 mg/L and 1 μg/L represent a toxicologically relevant concentration [23] and a predicted environmental concentration [24], respectively.

### 2.4. Chlorophyll Analysis

The chlorophyll content was measured following a method described by Wellburn and Lichtenthaler with slight modifications as described below [25]. The mixing suspension (4 mL) was centrifuged at 4000 rpm for 30 min. The algae cells were collected and added 4 mL of anhydrous ethanol in order to extract the chlorophyll. After 24 h reaction (4 °C) in the dark, the suspension was again centrifuged at 15,000 rpm for 10 min (4 °C) (using a D3024 High Speed Micro-Centrifuge; Scilogex, Rocky Hill, CT, USA). The supernatants were collected and stored for further studies. The optical density (OD) of the extracts at 663, 645 and 470 nm was measured for chlorophyll a, b and carotenoids. Chlorophyll contents were determined using empirical equations, as described in the Supplementary Data.

### 2.5. Mitochondrial Membrane Potential

A cationic fluorescent dye rhodamine 123 (Rh123, Aladdin) was used to measure mitochondrial membrane potential (MMP). Once the cells are equilibrated with the probe, depolarization (decrease in potential difference) will cause release of the dye into the medium and hyperpolarization (increase in potential difference) will cause uptake of the dye [26]. 96-h algal cell suspensions were centrifuged at 15,000 rpm for 10 min at 25 °C. Then the samples were incubated with 10 μM Rh 123 in the dark at 25 °C for 30 min, followed by washing three times with the algae medium.

### 2.6. Permeability of Cell Membrane

Fluorescein diacetate (FDA) purchased from the Aladdin Industrial Co. was used as a fluorescent probe to measure cell membrane permeability (CMP). Passive uptake of nonionic and nonfluorescent FDA by *S. obliquus* was measured by monitoring its intracellular hydrolysis, by nonspecific intracellular esterase, to produce the fluorescent molecule fluorescein [27]. 96-h algal cell suspensions were centrifuged at 15,000 rpm for 10 min at 25 °C. Then the samples were incubated with 10 μM FDA in the dark at 25 °C for 30 min, followed by washing three times with the algae medium.

### 2.7. Oxidative Stress Biomarker Assays

2′,7′-dichlorodihydrofluorescein diacetate (DCFH-DA) purchased from Macklin Biochemical Co., Ltd. (Shanghai, China) was used as a fluorescent probe to measure the intracellular ROS. The 96 h algal cell suspensions were centrifuged at 15,000 rpm for 10 min at 25 °C (using the D3024 high speed micro-centrifuge). After discarding the supernatant, 10 μM DCFH-DA was incubated with algal cells for 30 min under dark conditions at 25 °C. Subsequently, the samples were centrifuged under the same conditions and washed three times with the algae medium.

Superoxide dismutases (SOD), catalase (CAT), malondialdehyde (MDA) and total antioxidative capacity (TAC_SM_; the contribution of small molecules to antioxidant defenses) assays were performed using the appropriate commercial kits, which were purchased from Nanjing Institute of Jiancheng Biological Engineering (Nanjing, China). The test algae were cultured for 96 h and then harvested by centrifuging the medium (4000 rpm for 30 min). The precipitation was re-suspended in 1 mL phosphate buffer saline (PBS pH 7.2) and sonicated to break cells by an ultrasonic cell crusher (XO-650D, Xiaoou Tech, Nanjing, China). After sonication, the cell debris was removed by centrifugation at 15,000 rpm for 15 min at 4 °C (using the D3024 high speed micro-centrifuge) and the supernatant was used for biochemical assay. The assays were conducted according to the manufacturer’s instructions.

Superficial structures of *S. obliquus* exposed to the single, binary and ternary NPs were characterized by electron microscopy observation. 96-h algal suspensions were centrifuged at 4000 rpm for 1 h at 25 °C. After that, algal cells were chemically fixed for 2 h using 2.5% glutaraldehyde and then washed three times by PBS. Subsequently, the samples were dehydrated in a graded ethanol series (30%, 50%, 70%, 90% and 100% twice), washed with isoamyl acetate and all of the samples after each step above were centrifuged at 8000 rpm for 5 min at 4 °C. Finally, the samples dried under vacuum for 12 h. Images were obtained using the Hitachi S-4800 cold-cathode field-emission scanning electron microscope (SEM, Hitachi, Tokyo, Japan).

In this study, fluorescence intensity (FI) and absorbance were measured using a fluorospectrophotometer (F96PRO, Shanghai Kingdak Scientific Instrument Co., Ltd., Taizhou, Zhejiang, China) and an ultraviolet–visible spectrophotometer (UV1102; Shanghai Tian Mei Scientific Instrument Co., Shanghai, China), respectively. The excitation and emission wavelength for the optical measurements were previously described [26,27,28]. Data were expressed as a percentage (%) of the fluorescence or the absorbance of the control cells according to the equation:

%*F* = 100 − [100(*F*_c_ − *F*_t_)/*F*_c_] (1)

%*A* = 100 − [100(*A*_c_ − *A*_t_)/*A*_c_] (2)

where %*F* or %*A* is the percentage of fluorescence or absorbance of *S. obliquus* cells; *F*_c_ or *A*_c_, the mean fluorescence or absorbance of control cells; and *F*_t_ or *A*_t_, the mean fluorescence or absorbance of treated cells.

### 2.8. Statistical Analysis

All data are expressed as means ± standard deviation (SD). Statistically significant differences between the test treatments were determined by one-way analysis of variance at significance levels of *p* < 0.05, *p* < 0.01 and *p* < 0.001 (IBM SPSS Statistics for Windows, Ver. 19.0, IBM Corp., Armonk, NY, USA).

## 3. Results and Discussion

### 3.1. Physicochemical Characterizations

**Figure 1:**
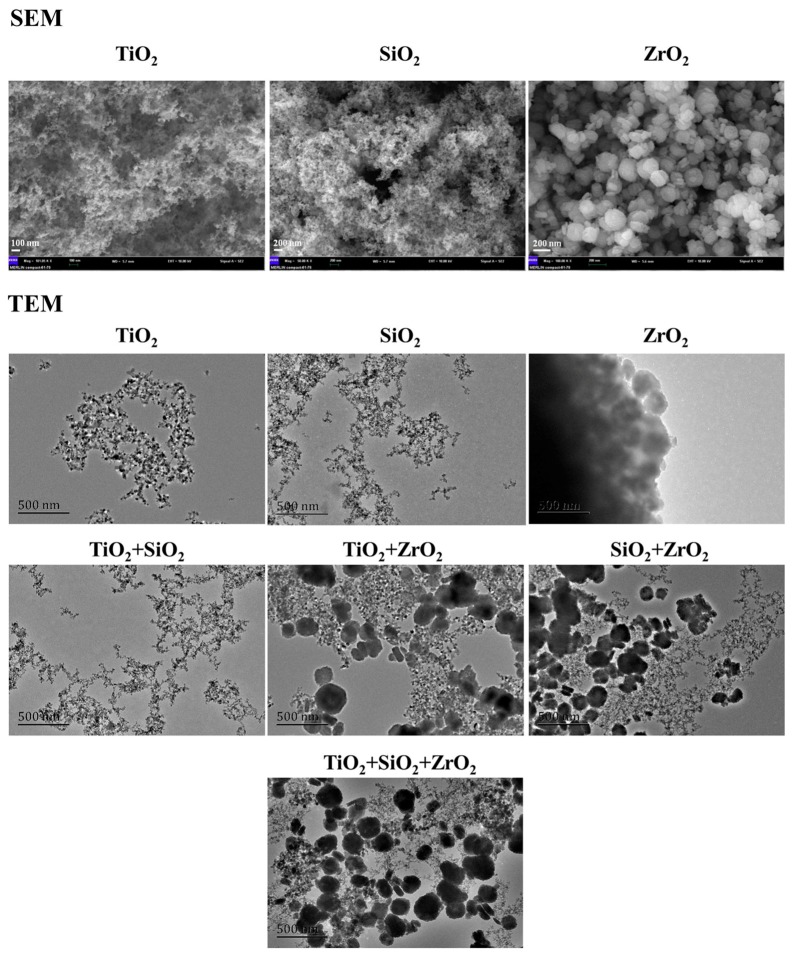
SEM images of the pristine NPs and TEM images of the NPs from single to ternary mixture systems in the algae medium.

The SEM and TEM images are depicted in Figure 1. The SEM images demonstrate that the individual TiO_2_, SiO_2_ and ZrO_2_ NPs used in this study were spherical particles. The primary diameters increased in the order SiO_2_ NPs (24.9 ± 5.4 nm) < TiO_2_ NPs (33.2 ± 6.2 nm) < ZrO_2_ NPs (247.8 ± 6.2 nm), which were higher than the advertised sizes. This implies that the TiO_2_, SiO_2_ and ZrO_2_ NPs aggregated. Moreover, the ZrO_2_ NPs showed greater tendency to aggregate than the other two NPs. The TEM images show the morphology of the studied NPs from single to ternary mixture systems in the algae medium (Figure 1). Analysis of the TEM images indicates that the NPs agglomerated intensely and formed irregular shapes in the test medium.

To evaluate the dispersion stability of the NP suspensions during the exposure, the size distribution and the surface charges of TiO_2_, SiO_2_ and ZrO_2_ NPs from single to ternary mixtures were determined in the algae medium (Table 1). In general, the ZP values of the ternary NP systems at 0 h were within the scope of the highest and lowest ZP values observed for the individual and binary NP systems. Over 96 h, the ZP values of the single NP systems and the binary TiO_2_ and SiO_2_/ZrO_2_ NP systems obviously increased, whereas the ZP values of the binary SiO_2_ and ZrO_2_ NP systems and the ternary NP systems showed no insignificant change. Similar to the finding of the ZP measurement, the *D*_H_ values of the ternary NP systems at 0 h were within the scope of the highest and lowest *D*_H_ values observed for the individual and binary NP systems. Over 96 h, the *D*_H_ values of the studied NP systems remarkably reduced, except for the single TiO_2_ and ZrO_2_ NP systems, as well as the binary SiO_2_ and TiO_2_/ZrO_2_ NP systems. These results suggest that the mixture of the NPs did not form colloidally stable complexes in the algal medium.

### 3.2. Effects of Single and Mixtures of NPs on Chlorophyll Contents

Chlorophyll is a major photosynthetic pigment for the photosynthesis of algae cells [29]. In order to observe the growth status of *S. obliquus*, we first determined the intracellular contents of chlorophyll a, b and carotenoids (Figure S1). In general, there was no significant difference in the chlorophyll contents in the tested groups, implying that the NPs alone and the combination of the NPs at the relatively low concentrations had no impacts on the activities of photosystems of the algae.

### 3.3. Effects of Single and Mixtures of NPs on Cellular Responses in Algal Cells

**Figure 2:**
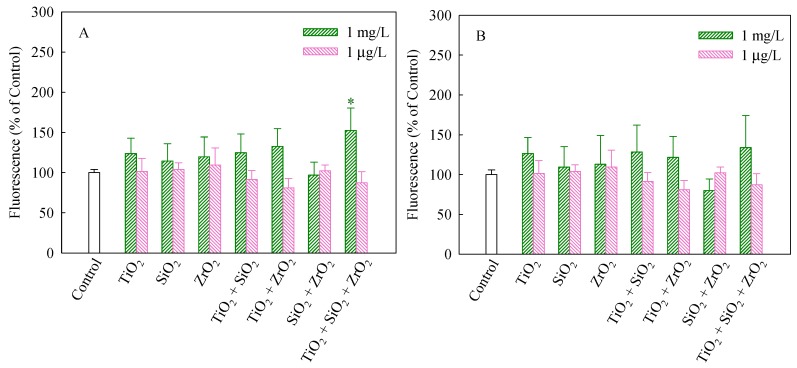
Mitochondrial membrane potential (**A**) and cellular membrane permeability (**B**) of *S. obliquus* exposed to 1 mg/L and 1 μg/L of NPs alone, 1 mg/L and 1 μg/L of single NPs in binary combination and 1 mg/L and 1 μg/L of single NPs in ternary combination. * indicates statistically significant difference from control (*p* < 0.05).

The stability of MMP and CMP is beneficial to maintain the normal physiological function of cells [26]. It has been suggested that mitotoxicity is a pathway for increasing oxidative stress [30]. As shown in Figure 2A, compared with the control group, only the ternary NP systems at the mixture concentration of 1 mg/L significantly increased MMP in the algal cells (*p* < 0.05) and thereby synergistically interfered with mitochondrial functions. The results shown in Figure 2B suggest that the tested groups had no significant impacts on CMP of *S. obliquus*.

**Figure 3:**
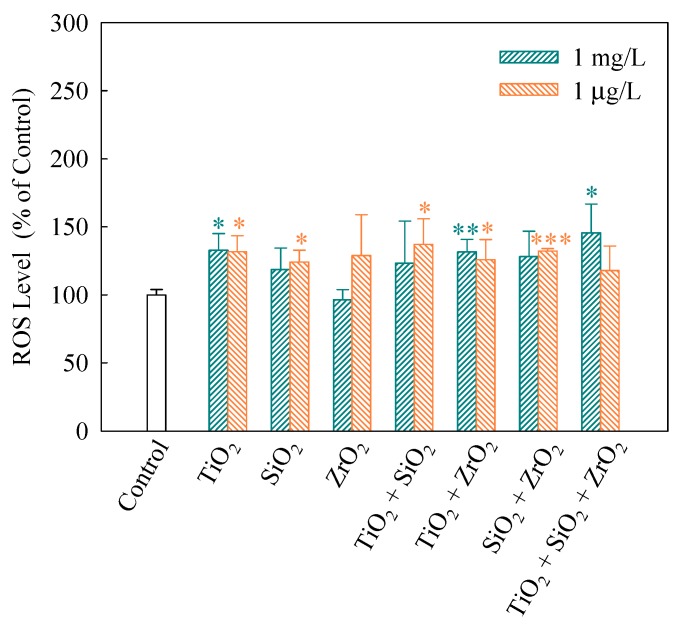
Relative levels of reactive oxygen species (ROS) detected using 2′,7′-dichlorodihydrofluorescein diacetate (DCFH-DA) staining in *S. obliquus* exposed to 1 mg/L and 1 μg/L of NPs alone, 1 mg/L and 1 μg/L of single NPs in binary combination and 1 mg/L and 1 μg/L of single NPs in ternary combination. Statistical significance versus control group: * *p* < 0.05, ** *p* < 0.01 and *** *p* < 0.001.

When intracellular ROS generate, 2′,7′-dichlorofluorescein (DCF) would be converted from DCFH, which was obtained by lipase decomposing DCFH-DA in cells [31]. Thus, FI of DCF indicates the extent of intracellular ROS generation. As shown in Figure 3, FI (%) of the ternary NP systems at the mixture concentration of 1 mg/L showed a significantly higher level (*p* < 0.05) than the control, indicating a significant increase in ROS. In addition, for the NP alone treatment, only TiO_2_ NPs significantly increased the ROS level. This implies that TiO_2_ NPs mainly contributed to the generation of ROS in the algal cells exposed to the ternary NP systems at the mixture concentration of 1 mg/L. Note that the binary NP systems at the mixture concentration of 1 μg/L significantly promoted the generation of intracellular ROS. Moreover, the individual TiO_2_ and SiO_2_ NPs at the concentration of 1 μg/L also accelerated an increase in the ROS levels in *S. obliquus*.

**Figure 4:**
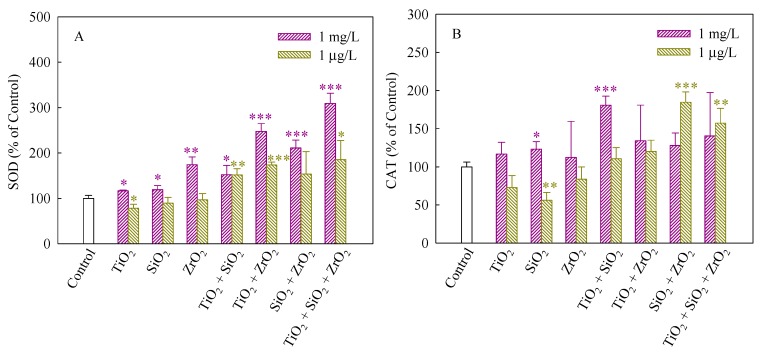
Activities of SOD (**A**) and CAT (**B**) in *S. obliquus* exposed to 1 mg/L and 1 μg/L of NPs alone, 1 mg/L and 1 μg/L of single NPs in binary combination and 1 mg/L and 1 μg/L of single NPs in ternary combination. Statistical significance versus control group: * *p* < 0.05, ** *p* < 0.01 and *** *p* < 0.001.

SOD and CAT can maintain steady-state levels of ROS in cells and protect cells against the adverse effects of them [32]. SOD dismutases superoxide anions (O_2_^−^) to hydrogen peroxide (H_2_O_2_), whereas CAT catalyzes the conversion of H_2_O_2_ to H_2_O [33]. Figure 4A,B depict the SOD and CAT activities of *S. obliquus* exposed to the NP suspensions from single to ternary mixture systems. As shown in Figure 4A, in comparison with the control, the SOD activity significantly increased when the algae were exposed to the single, binary and ternary NPs systems at the mixture concentration of 1 mg/L, demonstrating the production of O_2_^−^ and an increase in ROS scavenging. Generally, the up-regulation of SOD levels was consistent with an increase in ROS induced in response to exposure to the studied systems. Furthermore, the SOD activity induced by the ternary NP systems showed higher than the single and binary NP systems, suggesting that the three NPs exerted a synergistic influence on the SOD activity. In addition, the studied systems at the mixture concentration of 1 μg/L also significantly enhanced the SOD activity when TiO_2_ NPs were present, implying that TiO_2_ NPs in the mixtures contributed to the SOD activity mainly. In Figure 4B, CAT in treated cells exposed to 1 mg/L SiO_2_ NPs and the binary systems of TiO_2_ and SiO_2_ NPs at the mixture concentration of 1 mg/L was present significantly higher levels than the control. Moreover, the CAT activity of the binary systems of SiO_2_ and TiO_2_ NPs and the ternary NP systems at the mixture concentration of 1 μg/L was also higher than the control. However, the SiO_2_ NPs alone remarkably decreased the CAT activity, indicating that the activity of the enzyme might be inhibited and a decrease in the activity of CAT resulted in an inefficient removal of H_2_O_2_.

**Figure 5:**
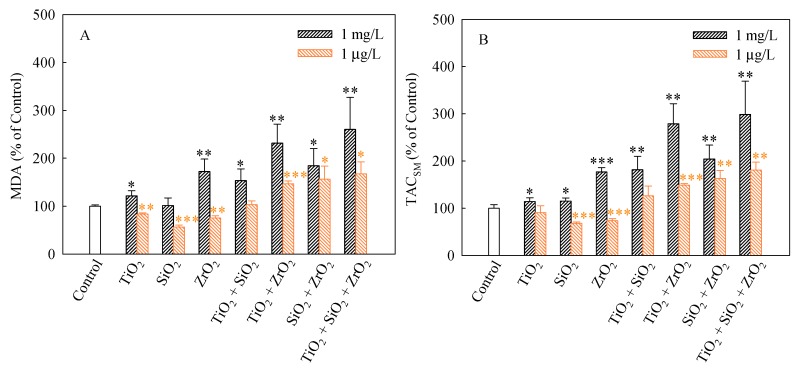
Levels of MDA (**A**) and TAC_SM_ (**B**) in *S. obliquus* exposed to 1 mg/L and 1 μg/L of NPs alone, 1 mg/L and 1 μg/L of single NPs in binary combination and 1 mg/L and 1 μg/L of single NPs in ternary combination. Statistical significance versus control group: * *p* < 0.05, ** *p* < 0.01 and *** *p* < 0.001.

We used MDA and TAC_SM_ as oxidative stress markers to evaluate the non-enzymatic antioxidant activities of *S. obliquus*. MDA production usually correlates with the occurrence of peroxidation [34]. MDA is considered to be one of the major products of lipid peroxidation under stressed conditions and it is widely used to determine the degree of oxidative stress that has occurred [35]. The MDA levels (%) in *S. obliquus* cells after 96 h of exposure to the studied systems are shown in Figure 5A. The levels of MDA in the algae cells exposed to the single, binary and ternary NP systems at the mixture concentration of 1 mg/L with the exception of SiO_2_ NPs were higher than the control. Furthermore, the MDA levels caused by the ternary NP systems showed higher than the single and binary NP systems. Additionally, the binary systems of TiO_2_ and ZrO_2_ NPs, the binary systems of SiO_2_ and ZrO_2_ NPs and the ternary mixtures at the mixture concentration of 1 μg/L induced a significant increase in the MDA levels. Moreover, the MDA levels in the algal cells exposed to these mixtures were obviously higher than the single NPs.

TAC_SM_ can reflect the contributions of small molecules, micronutrients, vitamins, and/or trace elements to antioxidant defense [36], which could be considered biomarkers of ROS levels in current studies [37]. As shown in Figure 5B, significant increases in the TAC_SM_ levels relative to the controls occurred in all the studied NP systems at the concentration of 1 mg/L. The TAC_SM_ levels also significantly increased in the binary systems of TiO_2_ and ZrO_2_ NPs, the binary systems of SiO_2_ and ZrO_2_ NPs and the ternary mixtures at the mixture concentration of 1 μg/L. It is noteworthy that the TAC_SM_ levels caused by the ternary NP systems showed higher than the single and binary NP systems. Hence it can be concluded that the ternary NP systems might result in more serious oxidative damage to *S. obliquus* cells. Moreover, particle-induced cellular responses depend on particle types and concentrations. This also means that increasing the types and the total number of NPs in multiple NP systems may enhance their toxicity in conjunction with other NPs.

**Figure 6:**
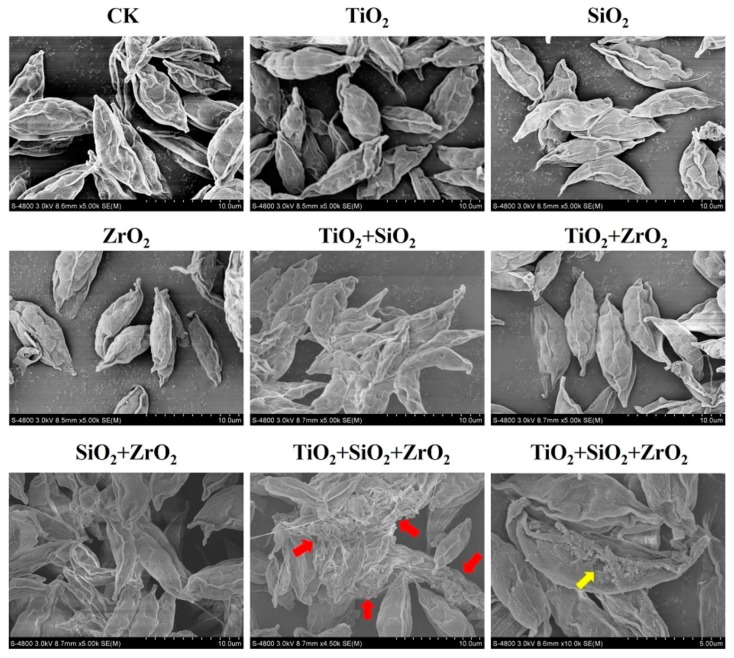
Superficial structures of *S. obliquus* exposed to 1 mg/L of NPs alone, 1 mg/L of single NPs in binary combination and 1 mg/L of single NPs in ternary combination. CK represents control. The red arrow and the yellow arrow indicate cell-cell adhesion and cell defect, respectively.

To further examine the findings from the biomarker assays, we observed the superficial structures of *S. obliquus* cells exposed to the single, binary and ternary mixtures of TiO_2_, SiO_2_ and ZrO_2_ NPs by the means of SEM (Figure 6). Compared with the control, the cells exposed to the ternary NP systems showed serious cell-cell adhesion. It is also noted that a structural defect in the cell surface was observed in a single cell when the ternary NP systems were present. Particle-mediated oxidative stress or ROS accumulation are considered likely mechanisms for the cell structure damage. Rather, compared with the control, no obvious structural change was observed for the individual NPs and their binary combination, suggesting that ROS induced by the single and binary NP systems might not be sufficient to activate the cell structure damage. Therefore, we further provide evidence that TiO_2_, SiO_2_ and ZrO_2_ NPs synergistically induced oxidative damage to the algal cells.

## 4. Conclusions

In conclusion, we have shown that TiO_2_, SiO_2_ and ZrO_2_ NPs at concentrations where individual NPs did not cause inhibition of photosynthesis in *S. obliquus* synergistically disturbed the mitochondrial membrane function, induced the cellular oxidative stress and triggered the enzymatic/non-enzymatic antioxidant defense systems. In-situ SEM observation indicates that the ternary mixtures of TiO_2_, SiO_2_ and ZrO_2_ NPs synergistically provoked oxidative damage to the algal cells. The types and the total number of NPs may contribute to the synergistic cellular response. Our findings highlight the importance of the synergistic toxicity assessment of the multiple NP combinations. To better understand the ecological risks of multiple NP systems, further study is needed to probe available methods for assessing and predicting the NP mixtures.

## Figures and Tables

**Table 1:** Zeta potential (ZP) and hydrodynamic diameter (*D*_H_) ± standard deviation of the test NPs from single to ternary mixture systems.

Test NPs	0 h		96 h	
	ZP	*D*_H_	ZP	*D*_H_
	mV	nm	mV	nm
TiO_2_	−20.9 ± 1.2	1111 ± 410	−15.3 ± 0.9	1278 ± 924
SiO_2_	−22.3 ± 1.6	525 ± 373	−17.9 ± 1.1	147 ± 38
ZrO_2_	−19.0 ± 0.5	333 ± 12	−16.3 ± 0.2	336 ± 41
TiO_2_ + SiO_2_	−16.7 ± 0.5	1095 ± 213	−13.8 ± 1.1	511 ± 319
TiO_2_ + ZrO_2_	−18.8 ± 1.0	596 ± 87	−16.3 ± 1.0	352 ± 22
SiO_2_ + ZrO_2_	−20.0 ± 0.5	327 ± 46	−19.8 ± 0.6	285 ± 30
TiO_2_ + SiO_2_ + ZrO_2_	−18.1 ± 0.2	523 ± 39	−18.5 ± 0.5	333 ± 10

## Supplementary Materials

The following are available online at http://www.mdpi.com/2079-4991/8/2/95/s1, Figure S1: Relative levels of chlorophyll a (A); chlorophyll b (B); carotenoids (C) and the total content of chlorophyll and carotenoids (D) in S. obliquus exposed to 1 mg/L and 1 μg/L of NPs alone, 1 mg/L and 1 μg/L of single NPs in binary combination, and 1 mg/L and 1 μg/L of single NPs in ternary combination.
