# Supplementary material for: TiO2, SiO2 and ZrO2 Nanoparticles Synergistically Provoke Cellular Oxidative Damage in Freshwater Microalgae
Source: Nanomaterials (Basel). 2018 Feb 8;8(2):95. doi: 10.3390/nano8020095 (PMC5853726; doi:10.3390/nano8020095)
Supplement: Supplementary file 1 [file nanomaterials-08-00095-s001.pdf]

Supplementary Materials

TiO_2, SiO_2 and ZrO_2 Nanoparticles Synergistically Provoke Cellular Oxidative Damage in Freshwater Microalgae

Yinghan Liu [1], Se Wang [1,*], Zhuang Wang [1,*], Nan Ye [1], Hao Fang [1] and Degao Wang [2]

[1] Collaborative Innovation Center of Atmospheric Environment and Equipment Technology, School of Environmental Science and Engineering, Nanjing University of Information Science and Technology, Nanjing 210044, China; yinghan_liu@163.com (Y.L.); yenan_nuist@163.com (N.Y.); fh@nuist.edu.cn (H.F)

[2] School of Environmental Science and Technology, Dalian Maritime University, Dalian 116023, China; degaowang@163.com

* Correspondence: wangse@nuist.edu.cn (S.W.); zhuang.wang@nuist.edu.cn (Z.W.); Tel./Fax: +86-25-58731090 (S.W. and Z.W.)

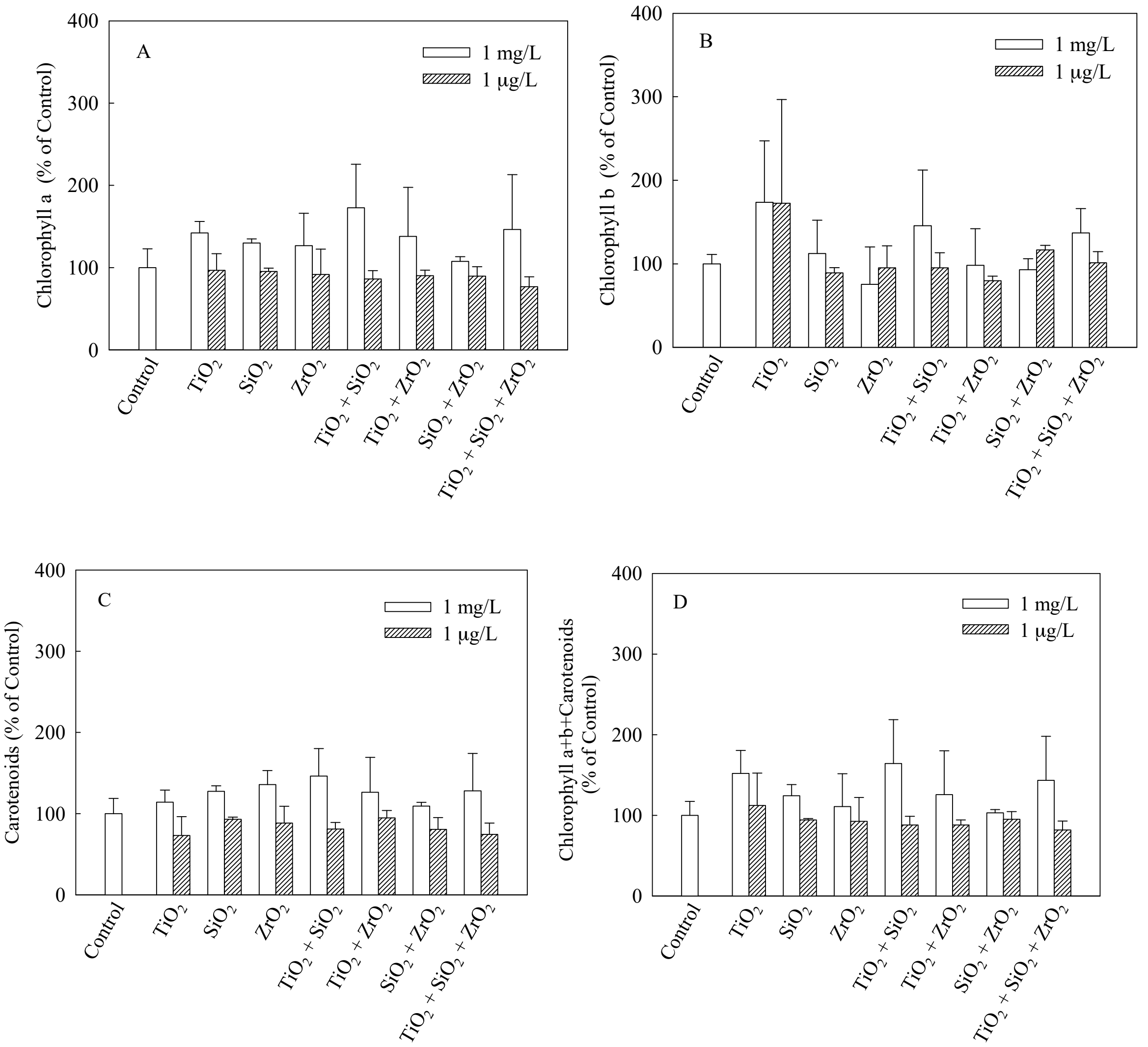

Figure S1. Relative levels of chlorophyll a (**A**); chlorophyll b (**B**); carotenoids (**C**) and the total content of chlorophyll and carotenoids (**D**) in *S. obliquus* exposed to 1 mg/L and 1 μg/L of NPs alone, 1 mg/L and 1 μg/L of single NPs in binary combination, and 1 mg/L and 1 μg/L of single NPs in ternary combination.

In the present study, chlorophyll contents were determined using the following empirical equations [S1]:

$$C_A = 12.21OD_{663} - 2.81OD_{645} \quad (1)$$

$$C_B = 20.13OD_{645} - 5.03OD_{663} \quad (2)$$

$$C_K = (1000OD_{470} - 3.27C_A - 104C_B)/229 \quad (3)$$

where C_A is the content of chlorophyll a, C_B the content of chlorophyll b, and C_K is the content of carotenoids [S2].

References

[S1] Wellburn, A.R.; Lichtenthaler, H. Formulae and program to determine total carotenoids and chlorophylls A and B of leaf extracts in different solvents. In: *Sybesma C*. Advances in photosynthesis research. Advances in agricultural biotechnology, Springer, Dordrecht, **1984**, *2*.

[S2] Li, X.; Ping, X.; Xiumei, S.; Zhenbin, W.; Liqiang, X. Toxicity of cypermethrin on growth, pigments, and superoxide dismutase of *Scenedesmus obliquus*. *Ecotoxicol. Environ. Saf.* **2005**, *60*, 188–192.
